# Exploring dog ownership in the lives of people with substance use disorder: a qualitative study

**DOI:** 10.1186/s13722-023-00411-z

**Published:** 2023-09-27

**Authors:** Andi Kerr-Little, Jørgen G. Bramness, Ruth C. Newberry, Stian Biong

**Affiliations:** 1 Norwegian National Advisory Unit On Concurrent Substance Abuse & Mental Health Disorders, Hamar, Norway; 2 Institute of Clinical Medicine, UIT The Arctic University of Norway, Tromsø, Norway; 3 Dept of Alcohol Drug and Tobacco Research, Norwegian Institute of Public Health, Oslo, Norway; 4 Department of Animal & Aquacultural Sciences, Faculty of Biosciences, Norwegian University of Life Sciences, Ås, Norway; 5 Lovisenberg Diaconal University College, Lovisenberggata 15B, 0456 Oslo, Norway

**Keywords:** Substance use, Recovery, Dog ownership, Qualitative methods, Content analysis

## Abstract

**Background:**

Recovery from substance use is commonly seen as a process of integrating social relationships and creating a sense of meaning in one’s life. Dog owners describe a close relationship with their dog that impacts many aspects of their everyday life. Yet for individuals with substance use disorder (SUD), little is known about how dog ownership could affect their lives. The aim of this study was to explore how people living with SUD experience and describe their everyday life when owning a dog.

**Method:**

Eight semi-structured in-depth individual interviews were conducted with people having personal experience of living with SUD and owning a dog. Data were gathered and analysed using qualitative content analysis.

**Results:**

The analysis yielded four categories, reflecting different aspects of dog ownership. Living with SUD and owning a dog was primarily something positive in their life. People increased their social connections personally and within society. They felt a belonging which gave a sense of agency and purpose, and they developed structure in their day and boundaries to their environment. Dog ownership, however, could hinder access to services which was found to be challenging for some participants.

**Conclusions:**

The owning of a dog can lead to changes that parallel those of a recovery process. This finding adds to the research on the connection that dogs can provide and shows how pertinent this can be particularly for vulnerable persons such as those with SUD.

## Introduction

Substance use disorder (SUD) is related to individual, social and structural factors. [1] Commonly marginalisation, social exclusion and stigmatisation are experienced [2, 3], along with challenges in accessing treatment and health services [4, 5].

Scientific knowledge about pet ownership amongst people living with SUD is scarce. Yet, people tend to form close bonds with their dogs, who are described to be as important as other family members [6–9]. Feelings of loneliness are reported to be reduced through pet ownership [10–12]. For people with mental health conditions; emotional support, social interaction and identity are all positive aspects of pet ownership [13, 14]. Dogs have been reported to increase meaningful community integration[15], and engagement in activities [16] for people with mental illnesses.

Although much of the research points to beneficial effects from relationships with companion animals, challenges can also arise. While pets were seen to provide emotional support and security similar to that of human attachment relationships [17], the complexities of one’s own attachment pattern can be reflected in the pet relationship [18], Difficulties also arise when the pet dies. This can lead to depression [19] and increased substance use [20], with those with a limited social network appearing to be most vulnerable [21]. Owning a pet can also prevent some people from leaving difficult situations such as domestic violence [22, 23]. Thus, the relationship with a pet can potentially amplify existing vulnerabilities.

In research focused on people experiencing homelessness, a comparable population in terms of having a reduced social network and high substance use [24, 25], pet ownership was associated with emotional well-being, love, trust, attachment and sense of purpose [26–31]. Pets were described as life savers [32] and the responsibility that comes with pet ownership was seen to potentially decrease destructive behaviours such as substance use, especially when provided alongside detoxification services [9, 20]. People living with homelessness and owning a pet also reported lower levels of depression and loneliness than non-pet owners [28, 30]. Challenges arose, however, with people experiencing homelessness foregoing overnight stays or health services due to no-pet policies [9, 27, 33].

Recovery from SUD is a transitional process away from substance use that can be understood in the context of everyday life [34, 35]. Comprising the personal and social dimensions involved in integrating relationships and meaningful activity [36, 37] the individual with their unique experience is considered the main decision maker in the process. [38, 39] A reduction in substance use comes alongside this recovery orientation rather than being the pivotal measure [34]. Conceptualising recovery within everyday life allows the significance of all of life’s intricacies and individual recovery outcomes to be included, even those so common that they are often unnoticed [40, 41].

One often overlooked yet meaningful relationship is that formed between people with SUD and their dog. As an area of growing interest, there are limited studies focusing on dog ownership for people with active substance use. However, dogs have been reported to promote a strengths-based approach to recovery for people on a methadone treatment programme [42] and pets have been identified as providing informal support for females in recovery [43]. Dogs have also been associated with benefits in exploratory dog-assisted interventions for people with SUD [44–48], and for veterans with post-traumatic stress disorder experiencing SUD [43].

In this qualitative exploratory study, we aimed to illuminate and gain a deeper understanding of how dog ownership is described and experienced by people in active substance use.

The research question was formulated as “How do people living with SUD experience and describe their everyday life when owning a dog?”.

## Methods

### Study design and population

Studying and generating knowledge from human experiences implies a qualitative approach. Since knowledge about pet ownership amongst people living with SUD is scarce, the design of the study was descriptive and exploratory. A collaborative approach with representatives from services working in the SUD field and a user representative who had personal experience of both dog ownership and substance use was engaged at different stages in the study process, i.e., including design of the study, recruitment strategy and analysis of the findings [49].

Recruitment and interviews took place in Oslo, Norway. Information pamphlets were handed out by staff at various low threshold centres [50], open user spaces, and through the assistance of outreach social workers. The first author (AKL) joined an outreach program to facilitate inclusion of people who were not normally in contact with services. No one who was interested in being interviewed was turned down though not all contacts lead to interviews.

The primary condition for inclusion was dog ownership whilst in active drug use. Current dog ownership was not a requirement, as it was felt that this allowed for a broader range of experiences and challenges to be heard. Exclusion criteria included children and others unable to give personal consent to participation in the study. A purposeful sampling strategy that aimed for a diversity of participants was applied [51].

Interviews were conducted with eight participants (Table 1). AKL (female, researcher with a therapeutic background) conducted the interviews. There was no prior relationship between AKL and the participants. AKL was introduced to the participants as a researcher for the study.

**Table 1:** Demographics of participants

Male/Female	Age (years)	Age started using substances (years)	What substance have you used most throughout lifetime	Where do you live	Do you live alone	What is your main income	Substitution treatment
Female	42	13	Heroin	Council accommodation	Yes	Benefits	Yes
Female	46	33	Heroin	Rented apartment	Yes	Benefits	Yes
Male	36	14	Cannabis and heroin	Council accommodation	Yes	Benefits	No
Male	50	14	Heroin and amphetamine	Rehabilitation centre	Yes	Benefits	Yes
Male	54	15	Hash, heroin, amphetamine	Council accommodation	Yes	Benefits	Yes
Female	40	16	Heroin	Homeless	Yes	Benefits	No
Female	53	15	Heroin	Council accommodation	Yes	Benefits	No
Female	48	9	Heroin	Council accommodation	Yes	Benefits	No

Six of the interviews took place in a quiet office at an open use centre, one occurred out of hours at an opiate maintenance centre and one took place in the participant’s own home due to anxiety about meeting elsewhere. If desired, the participants were given the opportunity to include their dog during their interview, and all but one of the participants who currently owned a dog brought the dog along. Pauses and breaks for dog interaction and care were included in the interview process. Participants had owned their dog from 1.5 years to 14 years, with an average of 5.9 years. Two of the participants were not current dog owners but had owned the dog previously.

### Data collection

As the actual knowledge base about pet ownership amongst people living with SUD is scarce, semi-structed, in-depth individual interviews were conducted [52]. This enabled direction yet allowed the participants freedom to expand upon their personal narratives around dog ownership. Follow-up questions were asked if something needed clarification or further explanation. The interviews took from 45 to 90 min. The interviews were audio recorded and transcribed verbatim by AKL. The participants received a gift voucher (200 NOK for their participation in interviews).

### Data analysis

Based on the study design and research question, a 4-step qualitative content analysis, focusing on the manifest content of the texts, was applied [53]. First, the transcripts were read through several times to enable a first impression of the text. This reading identified meaning units (i.e., a sentence or collection of sentences that contained a meaningful or central statement). The meaning units were then condensed and organised into codes (step 2). In Step 3, codes representing similar experiences were put together into subcategories, which were organized into related categories in step 4 (i.e., the manifest content in the texts). An example of the analysis process is included below (Table 2).

**Table 2:** Example of the analysis process from meaning units to category -Experiences of different relationships

Meaning unit	Condensed meaning unit (codes)	Subcategory	Category
“With a dog you meet other dog owners, not just dog owners, other people too that stop and, `how is the dog´, you get to know people, just like that, through the dog, and suddenly someone you meet maybe becomes part of your network and you get to know people, people have a need for a network, I am an older man, you know. It’s not so easy to find a network anymore as a 50-year-old man.”	Difficult to find a network. Through the dog, one meets dog owners and other people, who can be part of one´s network	Finding a network	You get to know people
“But you know, but it gives a responsibility both economically and physical, but to feel a part of society, and feel that you are part of the system, that’s what the dog does.”	The dog makes one feel part of society	Being part of society	
“I have two daughters. I think for them too it is good to know that I am not alone, that they can relate something positive to me and my life. They know I cannot be drug free but at the same time, there is a safety, as when they think about mamma it was just sad and drugs but now they know it’s not like that, now it’s [dog name] and walks in the forest.”	Before the dog, the daughters thought about mamma as alone, sad and unsafe. Now they relate something positive to mamma´s life	Creating safety and more positive relationships	

The analysis was done by AKL with a consensus on the categories developed through discussion with the other authors of the paper and the user representative.

## Results

Overall, owning a dog was described to have had a positive impact for the participants. The dog affected their lives in many ways, personal, social and practical. Four categories with subcategories were developed (Table 3).

**Table 3:** An overview of categories and subcategories

	Category		Sub-Category
1	You get to know people	1	Finding a network
		2	Being part of society
		3	Creating safety and more positive relationships
2	I couldn’t just let everything be messy around me	1	Closest relationship one has
		2	Unbreakable bond
		3	Something of worth in life
		4	Pet before self
3	He gives me fixed routines	1	She was employment for me
		2	Creating boundaries
		3	I had to calm down
		4	Challenge of economy
4	Because I had a dog, we cannot get accommodation	1	I am being punished
		2	I have to do it myself
		3	He is therapeutic for me

### You get to know people

This category represents how owning a dog provided a connection to other social relationships. The dog provided a social platform from which to talk to others, providing an ease of interaction that was hard to attain without a dog.

“With a dog, you meet other dog owners, not just dog owners, other people too [..] You get to know people, just like that, through the dog, and suddenly someone you meet maybe becomes part of your network and you get to know people. People have a need for a network. I am an older man, you know. It’s not so easy to find a network anymore as a 50-year-old man.” Male, age: 50.

Lives were described as isolated with few social connections. One man told of only having the pharmacy staff to talk to aside from his dog. Yet, participants described that having the company of the dog filled the space of lost social networks and helped them feel they could connect into society.

“…to feel a part of society and feel that you are part of the system, that’s what a dog does.” Male, age: 50

Interviewees also described the positive view others afforded them because of the dog. One man described how the dog acted as a facilitator in the broken relationship with his daughter. Another woman described how the dog helped her daughters gain a more positive view of her situation.

“I have two daughters. I think for them too, it is very good to know that I am not alone, you know, that they can relate something positive to me and my life, because they know I don’t manage to be drug free but at the same time there is a safety, as [before owning the dog] when they thought about mamma it was just sad and drugs, but now, they know it’s not like that. Now it’s [dog name] and walks in the forest.” Female, age: 48

However, not all connections related to dog ownership were viewed as positive. There were challenges described with others´ perceptions of their ability to care for a dog given their lifestyle and drug use. Although not a dominant perception, one of the participants described how animal welfare inspectors had been called to check the dog’s living conditions due to her substance use.

On balance, the dog was viewed as enabling relatively more positive engagement with society. The dog provided a bridge for connection to others and helped to mend broken family relationships.

### I couldn´t just let everything be messy around me

This category represents how owning a dog was associated with having a sense of belonging which gave meaning in life. Many of the participants moved from perceiving themselves as being alone to describing being part of something more than themselves. They belonged together with the dog, describing the dog as a family member, a partner, a baby or a child. They were not alone anymore. Their existence seemed to change when the dog arrived:

“Then all the focus was on him. I couldn’t just let everything be messy around me, you know. I had to make sure it was nice around me, and that did something to me, rather than everything just going over my head, you know. It was simply another focus that meant I also managed to take better care of myself, you know. He was so happy and playful—the care and love he gives, the being that he is, everything became much more positive. But no, it´s been a mix. The one takes care of the other, you know. To take care of him, then I take better care of myself.” Female, age: 48

The connection with the dog was described as unbreakable. Although there were many challenges in life, this relationship was resilient and trusted and was seldom seen as a difficulty. A sense of togetherness and belonging with the dog was a repeated theme for all participants. They were appreciative of having the dog in their life and felt that the dog made their life better.

Many of the interviewees described how caring for another had motivated them to be conscious of the choices in their life. Many described how they experienced a change in themselves due to having to consider the needs of the dog. Several participants described how this responsibility led to them putting the needs of the dog before their own needs. The dog gave them purpose in difficult times, the feeling that they were facing life together and that they could now feel that they had a future ahead of them.

“He helped me stay clean the whole way. After my father died […] I started using again but [dog’s name], he made it that my life was, it was something of worth because I had him.” Female, age: 46

### He gives me fixed routines

This category represents how owning a dog was connected to changes in daily life. The dog provided structure, something to get up for and set the daily rhythm.

“He gives me fixed routines and there is nothing better than that, and so I have obviously consistently stuck with it, first and foremost for him but also for myself.” Male, age: 54

Having this structure gave people motivation that was different from before they owned the dog. The day became organised and grounded with purpose. One man described how, after giving the dog up for rehoming, he found it difficult to get up in the morning. Another participant described how owning the dog gave him the motivation to ´sharpen up´ his life. Consistently, the participants described how the dog gave them something to do or ´employment´.

Owning a dog allowed participants to distance themselves from the drug scene. One participant described how, before she got the dog, she had had nowhere else to go, but after owning the dog she wanted to avoid the active drug environment. Another described how the dog enabled her to set boundaries, something she had previously found very difficult to do on her own.

“I think that he [the dog] has also helped me to set boundaries. For example, there are many places that I will never go to anymore because of him, and I set boundaries at home because he needs a very calm and predictable environment and I need that too […]. It’s much easier to set boundaries for others but this has become infiltrated in me too.” Female, age: 48

The participants also found that before owning a dog, their challenges with anxiety and depression could keep them isolated.

“I have always had anxiety, social anxiety, panic anxiety. There have been days when I just stood and looked out the living room window and had complete panic, because I had to go out [with the dog] for a walk, I had to just calm down, so she´s helped me so much.” Female, age: 40

Financial management was cited as a challenge in the daily life of pet owners. Some of the participants, however, had set up a savings account and described how they budgeted first for the dog, then for themselves. For others, saving and planning for times of need was seen as difficult. One participant described that the burden of financial responsibility meant that she felt forced to sell drugs to buy dog food.

“That was why I sold those pills. I have to go and buy food [for the dog] and I haven’t got any money.” Female, age: 46

Having the dog established a routine and a reason to set boundaries away from the drug scene. The financial aspect of dog ownership for some meant organising their economy, yet for others was challenging.

### Because I had a dog, we cannot get accommodation

This category represents how owning a dog was connected to practical issues in daily life and meeting with service providers. Requiring assistance from service providers was challenging as a dog owner. Accessing housing or temporary housing services was described as particularly difficult, giving rise to much anger, resentment and feelings of desperation.

“[The dog] has been Number One the whole time. Now I’m starting to cry. The worst it’s been was when we lost the apartment. It breaks my heart, oh God, that she doesn’t have a stable place to live. That’s what I think is tough, the toughest of all. I feel it is so painful […] when there is overnight accommodation available, but because I have a dog […] we cannot get accommodation. The situation is completely hopeless.” Female, age: 40

Others felt trapped regarding treatment services, expressing how they wanted to further their recovery process but could not do so because of the dog.

“It’s been a couple of years now and I’ve been hoping that I could come off [methadone]. I feel I may have to buy drugs and do it my own way. They don’t want me to do that—I should do it according to their rules, you know—but I feel I must do it that way as that’s the only choice I have been given because I can’t be away from him [the dog].” Male, age: 54

One man described how he had to give up his dog as there was no support available through a period of severe depression, although he considered the dog to be critical for his recovery process. If the service providers acknowledged the relationship with the dog, however, this created a positive connection and was regarded as a means whereby they felt understood.

“My doctor knows how much he means to me, so he wrote that this dog is a therapy dog for me. He is a therapist for me completely. He means so much to me.” Male, age: 36

## Discussion

The study aimed to give a voice to people with SUD and explore how owning a dog was perceived by them. Dog ownership impacted many areas of their lives, increasing social connections, creating a sense of belonging, and giving a stability and structure to their lives that had been lacking before. Yet challenges were apparent in meeting with service providers.

Owning a dog reduced the experience of marginalisation that is common among people with SUD. Integrating and feeling accepted in society can be challenging, yet as dog owners the participants described feeling ´part of the system´ and having increased connection. Often substance use is associated with high levels of stigma [2, 54] but, with a dog, these barriers seemed to lessen. These findings add to current literature on dog ownership acting as a social facilitator, building on the findings of [55] that pets increase a sense of community. Furthermore, in accordance with [56] and [57], our findings illuminate how dog ownership was able to unite people in personal relationships. The dog became the tie that mended family relationships broken by the individual’s substance use.

Taking steps to integrate into society is often thought to include employment or other means that provide structure and belonging. Several of the participants stated that owning a dog provided them with some of this same sense of accomplishment. Being useful and doing something of worth is seen to be important in recovery from comorbid disorders [58], particularly when this increases connection to society or nature [59]. In this study, the experience of dog ownership fulfilled some of these keys aspects, especially when employment was felt to be unattainable. Owning a dog also enhanced the associations and sense of belonging that the participants had outside of the substance use environment, thereby helping in distancing them from the identifying factors of SUD [60]. As [59] state, this helps in the transition from an addict identity to a recovery promoting identity. Such identity transition can be central in the recovery process [61–63], where recovery is thought of as a socially negotiated identity transition [64].

Davidson et al. [65] highlight the importance of positive life events and the inclusion of often overlooked everyday occurrences in the recovery journey. With the dog, life became fuller and more anchored with meaning, described as ´having something of worth in their life´ allowing the participants to gain agency in their lives. They actively made choices regarding their daily life and found grounds for maintaining their personal boundaries. This sense of agency and meaning is aligned with recovery from mental health conditions, where a strengths-based approach is utilised to allow for the restoration of meaning despite or alongside the condition [66]. The accumulation of these everyday changes’ fosters what [67] refers to as a recovery-friendly environment.

On a personal level, the dog was often described as a family member, with whom the participant had an unbreakable bond. The relationship with the dog was described as unconditional and supportive. This is in contrast to a lack of close relationships and high levels of loneliness in interpersonal relationships often reported by people with SUD. [68] This lack of personal connection can be a risk factor leading to a vicious cycle of substance use. [69, 70, 70] state the significance of intimate positive relationships in long term recovery, highlighting the importance of being able to feel close to others without feelings of shame or guilt. Yet, as vital as interpersonal relationships are, people who suffer from substance use often report complexities regarding intimate relationships. [72] In this study and in accordance with [73], the relationship with the dog appeared to ease the obligations and complexities associated with other close relationships and provided a connection that was trusted, safe and secure.

For some, ownership of the dog highlighted the fragility of their situation, namely the need to sell drugs to secure money for dog food. This was a worrying finding as those with SUD tend to be vulnerable to life’s challenges and for one participant, the additional responsibility of dog ownership added to an already precarious situation. For this participant, the responsibility of dog ownership appeared to be at the limit of what they could cope with. This raises concerns that the welfare of the dog, and the feelings of accomplishment gained through dog ownership, may be jeopardised if an individual has a downward period. Feelings of shame and failure tend to be characteristics easily exacerbated in people with SUD which can escalate a tendency towards substance use. [74]

Access to housing and services as a dog owner was deemed particularly challenging. How the dog was viewed by the service providers impacted the perceived trust and support felt by the participants. At times within SUD, help is sought but the process of accessing treatment or other services falls short due to unknown obstacles [75]. This study indicates that dog ownership could be one of these obstacles. Access to housing services is essential in moving people away from a marginalised state, yet the participants described this as particularly challenging due to limited pet-friendly options. Building positive connections with service providers was seen as essential in long term recovery from substance use [71]. Thus, recognising the pet relationship could be a potentially under-used resource.

From the present study, it appears that the relationship with the dog confers many benefits to the owner, both on a personal and societal level. Yet there are pitfalls in terms of accessing services. These findings add to the growing literature on the importance of human-animal relationships in mental health. This study highlights how a close interpersonal relationship with a dog gives depth and meaning which transfers to other areas of life and community. The importance of considering an individual with SUD in the context of their life and embedded social environment, be that human or animal, is emphasised. Often, the relationship with the dog created a connection that human relationships had not been able to do, and initiated changes that paralleled recovery, although out-with a formal recovery programme. A tailored personalised approach is required from service providers, as owning a dog appears to be a potentially promising resource that has until now been scarcely considered. The current findings are in accordance with the main theories on human-animal interaction, whereby owning a pet provides a sense of belonging through attachment and social facilitation.

### Methodological strengths and limitations

Qualitative studies with a narrative approach can shed light on how the phenomenon under study is experienced by humans. This approach allowed us to gain an enhanced understanding of the phenomenon of dog ownership among people living with SUD, which may be translated to other contexts and generate hypotheses and further studies in an area that has until now been largely overlooked.

Nevertheless, the analytic approach in this study had a limited capacity for exploring processes over time and the results reflect the experiences of a limited sample of people living with SUD and owning a dog in a major city in a Nordic welfare state. The study was based on eight qualitative interviews, with the number restricted by challenges in the scheduling of interviews in this population and the potential burden on them of a long interview. We believe that the participants´ rich descriptions were detailed enough for us to be able to answer our research question. Drawing on the work of [76, 77], an assessment was made weighing the sensitivities of the participant groups against the data gathered to decide when enough interviews had been conducted.

The sampled participants all seemed to have a good relationship with their dog and consisted of people who were willing and able to participate. Inclusion of additional participants might have led to the illumination of additional negative as well as positive experiences associated with dog ownership. The study also did not differentiate between possible differences related to age and gender.

## Conclusions

This study showed that participants living with SUD experienced dog ownership as something primarily positive in their life. People increased their social connections both personally and within society, they felt they belonged to something which gave a sense of agency and purpose within themselves, and they developed a structure to their day and boundaries within their environment.

Understanding the importance of the relationship with a dog for people with SUD can help to build connection where patterns of disconnect exist. This study also sheds light on how the process of recovery can emerge out of one’s own life progression and emphasises the importance of seeing the individual within their entire embedded network, including companion animals.

Dog ownership, however, did not come without challenges, with participants experiencing difficulties in accessing services and, at times, with the daily structuring of dog ownership.

## Data Availability

The datasets used and/or analysed during the current study are available from the corresponding author on reasonable request.
